# A Putative Plant Aminophospholipid Flippase, the Arabidopsis P4 ATPase ALA1, Localizes to the Plasma Membrane following Association with a β-Subunit

**DOI:** 10.1371/journal.pone.0033042

**Published:** 2012-04-13

**Authors:** Rosa L. López-Marqués, Lisbeth R. Poulsen, Michael G. Palmgren

**Affiliations:** Department of Plant Biology and Biotechnology, Centre for Membrane Pumps in Cells and Disease (PUMPKIN), University of Copenhagen, Danish National Research Foundation, Frederiksberg, Denmark; University of South Florida College of Medicine, United States of America

## Abstract

Plasma membranes in eukaryotic cells display asymmetric lipid distributions with aminophospholipids concentrated in the inner leaflet and sphingolipids in the outer leaflet. This unequal distribution of lipids between leaflets is, amongst several proposed functions, hypothesized to be a prerequisite for endocytosis. P4 ATPases, belonging to the P-type ATPase superfamily of pumps, are involved in establishing lipid asymmetry across plasma membranes, but P4 ATPases have not been identified in plant plasma membranes. Here we report that the plant P4 ATPase ALA1, which previously has been connected with cold tolerance of *Arabidopsis thaliana*, is targeted to the plasma membrane and does so following association in the endoplasmic reticulum with an ALIS protein β-subunit.

## Introduction

The plasma membrane (PM) is the outermost barrier of a cell and is responsible for the exchange of matter and information between its exterior and the interior. First recently it has been acknowledged that the asymmetric distribution of lipids between the two leaflets of the plasma membrane is important for its proper function. In animal cells, the aminophospholipids phosphatidylserine and phosphatidylethanolamine (PE) are in general sequestered in the inner leaflet of the plasma membrane, whereas sphingomyelin and glycosphingolipids are enriched in the outer leaflet [1], [2]. Amongst other features, asymmetric transversal distribution of phospholipids, sphingolipids, and free sterols between the cytosolic and apoplastic (exoplasmic) leaflets is required for maintaining a low permeability to solutes [3], [4].

Among lipids, phosphatidylserine in particular is observed to distribute unevenly between the two leaflets [5]–[7]. The phosphatidylserine asymmetry and its regulation is considered important to cellular processes such as cell-cell recognition [8], endo- and exocytosis [1], regulation of membrane surface potential [7] and activation of membrane-bound enzymes [8], [9] as indicated in mammalian and yeast cells. Regulated loss of this asymmetric lipid arrangement triggers a variety of physiological events including apoptosis [10].

In the yeast *Saccharomyces cerevisiae* the plasma membrane phospholipid asymmetry is influenced by ATP-dependent aminophospholipid translocases which constitute a subfamily, P4 ATPases, in the superfamily of P-type ATPase pumps [11]–[13]. *S. cerevisiae* has five P4 ATPases, among which two are localized in the plasma membrane, namely Dnf1p and Dnf2p [14]. Loss of Dnf1p and Dnf2p leads to an increased cell surface exposure of endogenous phosphatidylserine, which is enhanced by additional removal of the Golgi-localized P4 ATPase Drs2p [15]. Concurrent with an altered phospholipid arrangement in the plasma membrane, *Δdrs2Δdnf1Δdnf2* cells exhibit a defect in the uptake of the endocytic tracer FM4–64 and in the ligand-induced internalization of α-factor receptor [14]. These results point to a functional link between P-type ATPase-dependent lipid translocation and budding of endocytic vesicles from the plasma membrane.

P4 ATPases from protozoa and animal cells have also been localized to the plasma membrane and shown to be involved in phospholipid translocation across this membrane. These include *Leishmania donovanii* LdMT, responsible for transporting the drug miltefosine, a toxic choline ether lipid used in treatment of the leishmaniasis disease [16], [17], human ATP8B1, involved in a severe liver disease in humans [18], and mouse FetA, involved in formation of the acrosomal membrane in sperm cells [19]. In addition to being involved in phospholipid flipping [18], ATP8B1 has a structural or signalling role in formation of microvilli in intestinal cells, which appears to be independent on lipid transport across the plasma membrane [20].

In plants, much less is known on the influence of lipids on the functions of plasma membranes. It is generally assumed that a transversal lipid asymmetry exists also in plant plasma membranes [21], [22], but the only analysis so far conducted on plant material concluded that phosphatidylserine is the only phospholipid asymmetrically distributed between the plasma membrane leaflets [23]. The physiological significance of the phosphatidylserine asymmetry in plant plasma membranes is still unclear, and the existence of phospholipid flippases in plant plasma membranes has not yet been shown. Takeda and Kasamo [23] tried to detect phospholipid flippase activity in the inside-out plasma membrane vesicles created by Brij 58-treatment using (oleoyl-C12-NBD)-phospholipids under various conditions, but could not find such an activity, although phosphatidylserine was concentrated in the inner leaflet.

In the model plant Arabidopsis, 12 P4 ATPase genes are present [24]. Recently, we have demonstrated that two Arabidopsis P4 ATPases ALA2 [25] and ALA3 [26], localize to the prevacuolar compartment (PVC) and the Golgi apparatus, respectively, and require a β-subunit (ALIS protein) for exit from the endoplasmic reticulum (ER) and for transport of phospholipids. A third Arabidopsis P4 ATPase, ALA1, has been characterized and shown to be able to transport lipids in yeast in the absence of a co-expressed plant β-subunit [27]. However, the subcellular localization of the protein was never investigated. In this work, we demonstrate the ALA1 localizes to the plant plasma membrane and has a strict requirement for a β-subunit to exit the ER.

## Results

### ALA1 is Retained in the ER in the Absence of an ALIS Protein

**Figure 1:**
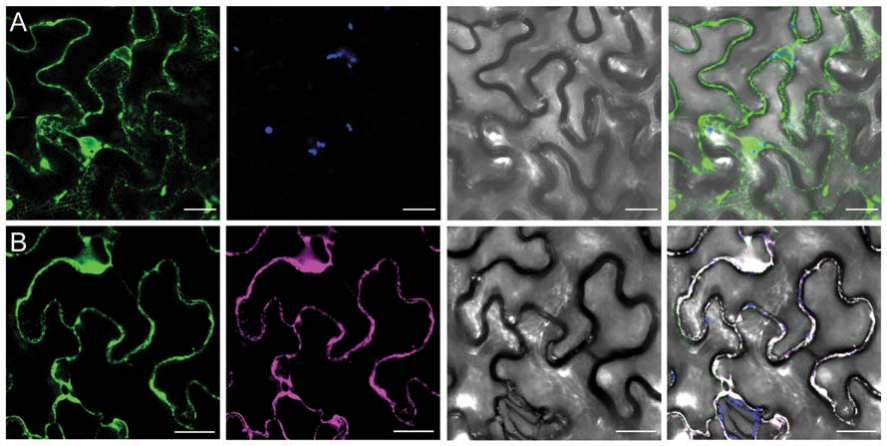
ALA1 expressed without a β-subunit localizes to the ER in planta. A *GFP::ALA1* fusion was transiently expressed in tobacco epidermal cells alone (**A**) or in the presence of an YFP::HDEL ER-retained marker (**B**). *Green*: GFP fluorescence; blue: autofluorescence; *magenta*: YFP fluorescence; *gray*: bright field image. Panels on the *right* show an overlay image. Scale bar: 25 µm.

Transient expression in tobacco epidermal cells has been used before to demonstrate that Arabidopsis P4 ATPases are retained in the ER in the absence of an ALIS protein [25], [26]. In order to express and visualize ALA1 in tobacco, the genomic DNA fragment corresponding to this protein was cloned and placed under the control of its own promoter in a plant binary plasmid containing an in-frame fusion with Green Fluorescent Protein (GFP). However, this construct did not generate a detectable fluorescent signal when infiltrated in tobacco cells. To overcome this problem, the *ALA1* genomic DNA was re-cloned into plasmids of the pMDC series [28], which contain a double 35 S promoter and allow for fusion of a GFP at each end of the protein of interest. Both the N- and the C-terminally tagged *ALA1* gDNA constructs presented a clear fluorescent signal in membrane structures that resembled the ER (Figure 1A and not shown). In order to confirm the nature of these membranes, the ALA1 fusions were co-expressed with a construct containing a Yellow Fluorescent Protein (YFP) modified to include an HDEL ER retention signal at the C-terminal end (Figure 1B). Co-localization of both fluorescent proteins confirmed that ALA1 resides in the ER membrane when expressed alone. Under our experimental conditions, no bleed-through of the fluorescence signals was detected (see Figure S1).

### ALA1 Leaves the ER and Localizes to the Plasma Membrane in the Presence of an ALIS Protein

**Figure 2:**
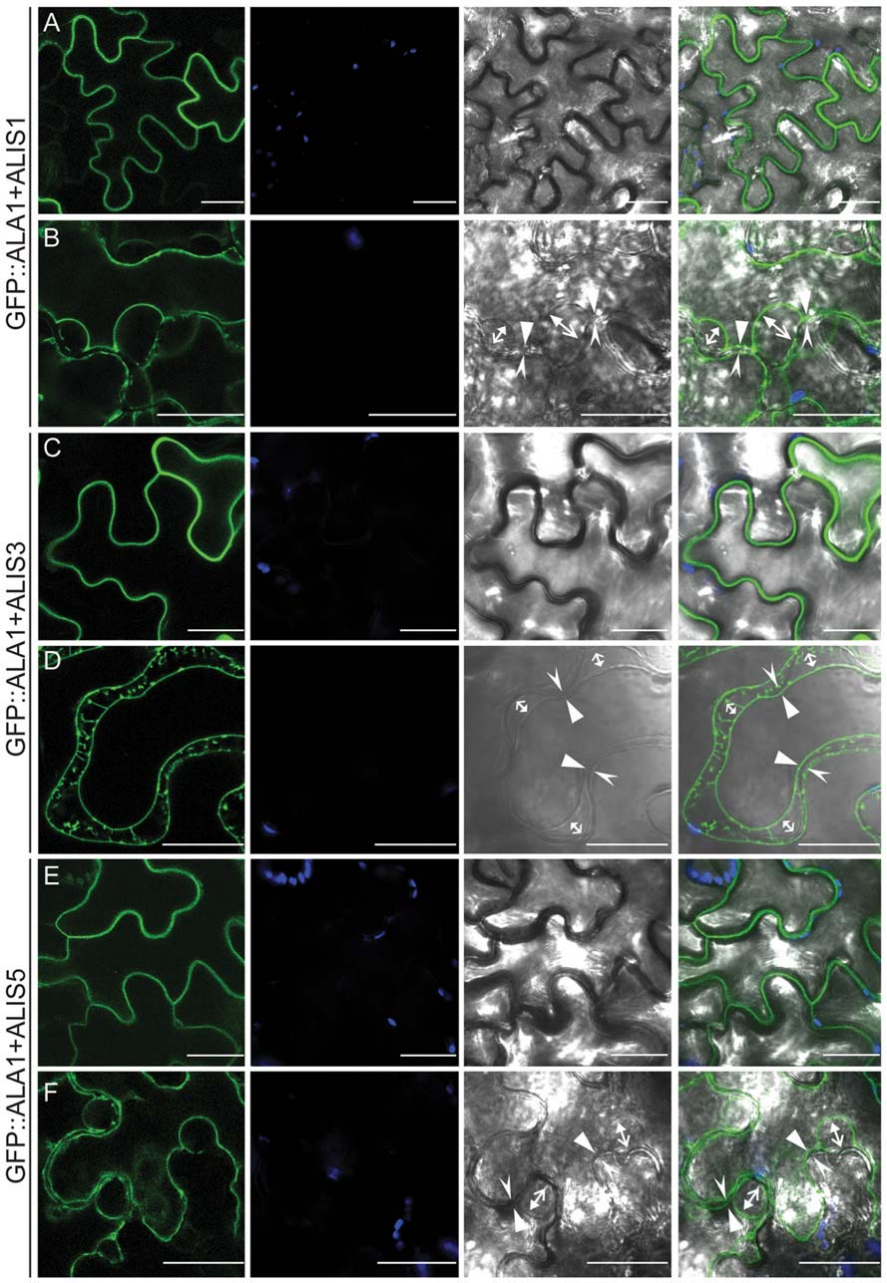
ALA1 localizes to the plasma membrane in the presence of an ALIS protein. A *GFP::ALA1* fusion was transiently expressed in tobacco epidermal cells in the presence of an untagged ALIS. (**A,B**) ALIS1; (**C,D**) ALIS3; (**E,F**) ALIS5. (**A,C,E**) untreated cells. (**B,D,F**) cells plasmolyzed in the presence of a concentrated solution of manitol. *Arrows* in the bright-field and overlay images indicate the relative positions of the plasma membrane and the cell wall in points at which they have detached (opposing arrows) or remain attached (facing arrows) during plasmolysis. The plasma membrane is indicated with a closed arrow and the cell wall with open arrows. *Green*: GFP fluorescence; *blue*: autofluorescence; *gray*: bright field image. Panels on the *right* show an overlay image. Scale bar: 25 µm.

In order to investigate whether ALA1 is an ER-resident protein or whether it leaves the ER upon interaction with an ALIS β-subunit, we transiently expressed the N-terminally GFP-tagged ALA1 fusion in tobacco leaves in the presence of untagged versions of three different ALIS proteins (ALIS1, ALIS3, ALIS5) [26]. Co-expression with any ALIS protein resulted in localization of the GFP fluorescence in membranes resembling either the plasma membrane or the tonoplast (Figure 2A,C,E). In tobacco epidermal cells, the vacuole occupies almost al the volume of the cell, pressing all other organelles against the rigid cell wall. Thus, under normal conditions, it is not possible to distinguish the plasma membrane from the tonoplast, as they both run along the cell wall. However, while the tonoplast can freely shrink under hyperosmotic conditions, the plasma membrane is attached to the cell wall at several contact points along the surface of the cell [29]. Thus, plasmolysis of the cells by addition of a hyperosmotic solution allows distinguishing between the two membranes. When the cells were plasmolyzed in the presence of a hyperosmotic mannitol solution, several detachment areas flanked by attachment points to the cell wall could be seen for the GFP-containing membranes, which is to be expected for a plasma membrane localization (Figure 2B,D,F). The same result was obtained for an ALA1::GFP fusion (not shown).

### ALIS Proteins are Localized to the Plasma Membrane When Co-expressed with ALA1

**Figure 3:**
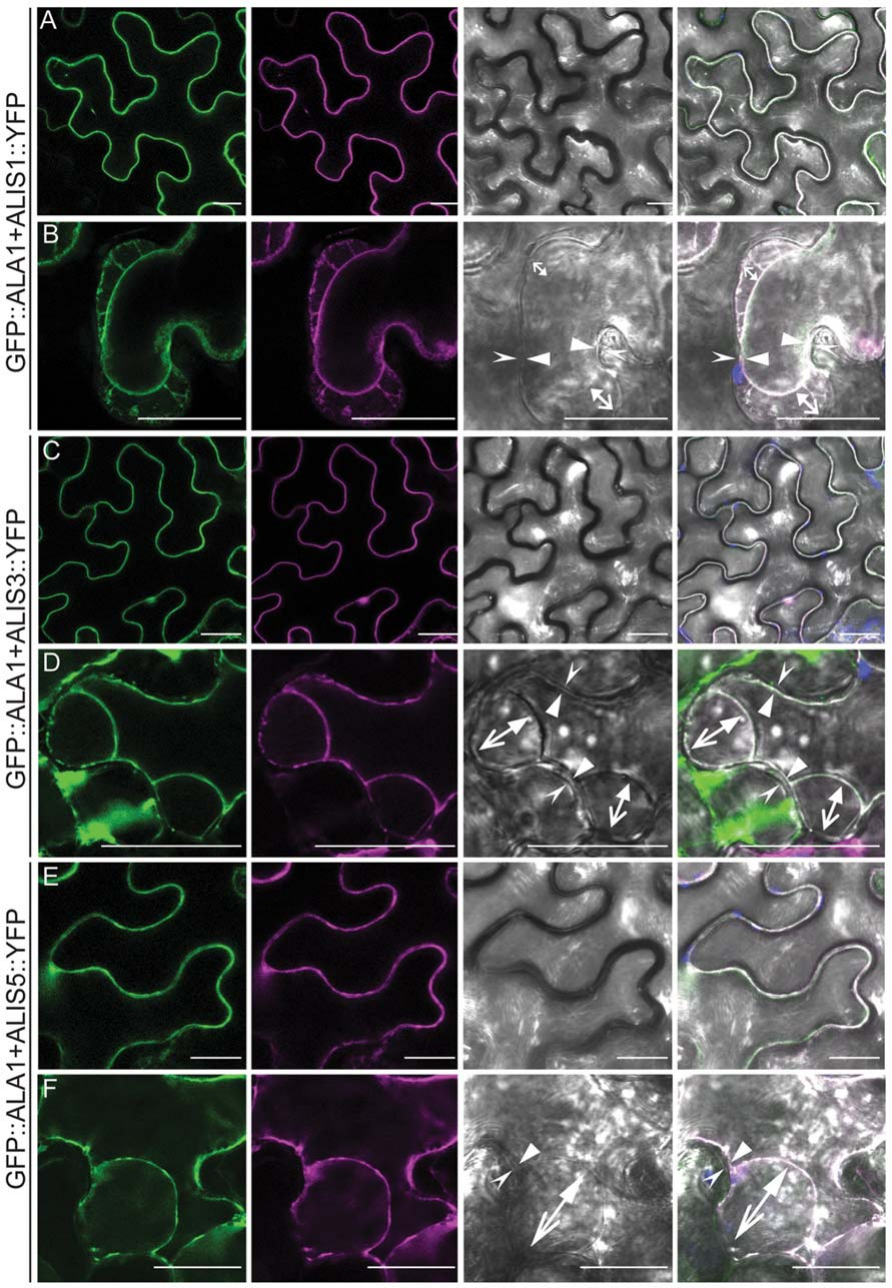
ALIS proteins co-localize with ALA1 to the plasma membrane. A *GFP::ALA1* fusion was transiently expressed in tobacco epidermal cells in the presence of YFP-tagged ALIS (*ALIS::YFP*). (**A,B**) ALIS1; **C,D**: ALIS3; **E,F**: ALIS5. **A,C,E**: untreated cells. **B,D,F**: cells plasmolyzed in the presence of a concentrated solution of manitol. *Arrows* in the bright-field and overlay images indicate the relative positions of the plasma membrane and the cell wall in points at which they have detached (opposing arrows) or remain attached (facing arrows) during plasmolysis. The plasma membrane is indicated with a closed arrow and the cell wall with open arrows. *Green*: GFP fluorescence; *magenta:* YFP fluorescence; *gray*: bright field image; *blue*: autofluorescence. Panels on the *right* show an overlay image. Scale bar: 25 µm.

Previously we have demonstrated that the localization determinants for the ALA/ALIS complex reside in the α-subunit and that ALIS proteins are localized to the same compartment as the ALA protein they are co-expressed with. This was shown both for Golgi-localized ALA3 [26] and for the prevacuolar compartment-resident protein ALA2 [25]. To investigate if this was also the case for plasma membrane-localized ALA1, C-terminally YFP-tagged versions of ALIS1, ALIS3 and ALIS5 [26] were co-expressed with GFP::ALA1 (Figure 3). In all cases, the fluorescent signals co-localized to membranes resembling the plasma membrane. As above, plasma membrane localization could be confirmed by plasmolysis of the cells.

### Heterologous Expression of ALA1 in a *Δdrs2Δdnf1Δdnf2* Yeast Mutant Strain

Heterologous expression of plant proteins in the host *S. cerevisiae* is a common tool to obtain protein for functional analysis and to test whether gene expression in this host can functionally complement mutations in related yeast genes. For this purpose, a cDNA fragment codifying for haemagglutinin (HA)-tagged ALA1 was cloned alone or together with Arg-Gly-Ser-His_6_ (RGSH6) tagged ALIS1, ALIS3 or ALIS5 into yeast plasmids that contain a bi-directional *GAL1-10* promoter, which allows for expression of two proteins at the same time, and introduced into a *Δdrs2Δdnf1Δdnf2* yeast mutant strain.

**Figure 4:**
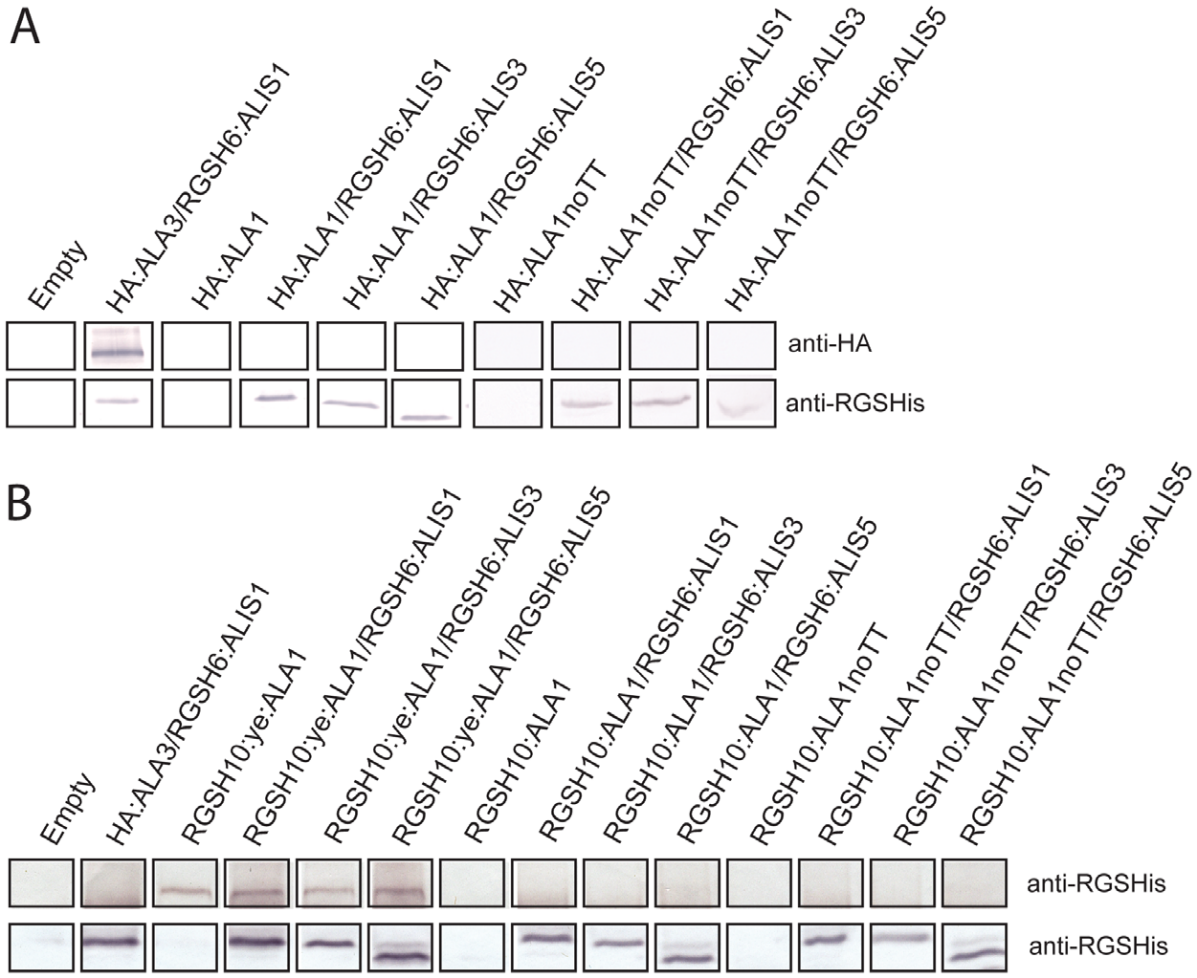
Expression of ALA1 in yeast requires optimization of the cDNA sequence. (**A**) Total membrane protein preparations (30 µg) were run on SDS-PAGE gels and subjected to immunodetection with antibodies raised against the engineered epitope tags. Only ALIS proteins and yeALA1 can be detected with the anti-RGSH antibody. (**B**) *ALA1* cDNA was modified to eliminate a putative transcription termination site and five rare codons codifying for Arg (yeALA1) and expressed alone or together with an ALIS in a yeast *Δdrs2Δdnf1Δdnf2* deletion mutant. ALA1 was tagged at the N-terminal end with an RGSH10-tag while ALIS proteins bear a RGSH6-tag at the same end. *ALA1* and *ALA1noTT* (*ALA1* cDNA codified to eliminate a putative transcription termination site) were also expressed under the same conditions. Total membrane protein preparations (30 µg) were run on SDS-PAGE gels and subjected to immunodetection with antibodies raised against the engineered epitope tags. Bands corresponding to ALIS proteins are immunodecorated with an anti-RGSH antibody, while an anti-HA antibody cannot recognize any band corresponding to HA-tagged ALA1.

To verify expression of ALA1 in yeast, we took advantage of the epitope tags engineered at the N-terminal end of the proteins. Immunodetection in total yeast membranes with antibodies raised against the HA or the RGSH6 epitopes showed that ALA1 is not expressed to detectable levels (Figure 4A), while clear signals could be seen for HA-tagged ALA3 (used as a positive control) and for all RGSH6-tagged ALIS proteins. In order to increase the level of expression, we searched for genetic elements in the introduced cDNA that could possibly affect negatively heterologous expression in yeast and identified a putative transcription termination signal located about 300 amino acid residues before the stop codon of ALA1 (see Figure S2). Using an overlapping PCR strategy, this transcription termination site was removed from the cDNA sequence. The new ALA1 version, ALA1noTT, was expressed in yeast, both in untagged or tagged versions, alone or together with RGSH6-tagged ALIS1, ALIS3 and ALIS5. Following this approach, the HA-tagged version of ALA1noTT was also not detectable in total yeast membranes (Figure 4A).

Next, we used a graphical codon usage analyser to identify rare codons that might result in low levels of expression of ALA1 in *S. cerevisiae*. Based on the results of this analysis, five codons codifying for arginine were mutated from CGG to AGA or AGG, generating a yeast-enhanced ALA1 (yeALA1) (Figure S2). Simultaneously, we aimed at increasing expression levels by modifying the *GAL1* galactose-inducible promoter contained in our yeast plasmids to include the leader sequence from the strong yeast *PMA1* promoter. A RGSH10 tag was also included for immunodetection of expressed proteins. A new construct containing *yeALA1* under the control of the modified promoter was transformed into *Δdrs2Δdnf1Δdnf2* together with plasmids either empty or containing a RGSH6-tagged ALIS1, ALIS3 or ALIS5 fusion. Following isolation of yeast microsomes, tagged proteins representing ALA1 as well as ALIS1, ALI3 and ALIS5 could now be clearly detected in protein blots (Figure 4B).

In order to compare all versions of the *ALA1* gene expressed from the same plasmid and bearing the same tag, homologous recombination in yeast was used to generate plasmids containing the cDNA fragments codifying for ALA1 and ALA1noTT under the control of the *GAL1-10* promoter::*PMA1* leader fusion. Both constructs were transformed into *Δdrs2Δdnf1Δdnf2* together with plasmids either empty or containing RGSH6-tagged ALIS1, ALIS3 or ALIS5, as above. In this case, no ALA1 protein could be immunodetected by western blot (Figure 4B), indicating that, under our experimental conditions, modification of the codon usage for Arg in yeALA1 was crucial for obtaining detectable levels of expression for the protein.

In previous studies, we noted that under our working conditions a single *Δdrs2* yeast mutant shows a high incidence of genetic revertants when tested for growth at temperatures around 20°C, while a *Δdrs2Δdnf1Δdnf2* strain is a more reliable system for testing functional complementation of the *drs2* related cold-sensitive phenotype [26]. Moreover, translocation of natural lipids by heterologously expressed P4 ATPases can be tested in this triple mutant strain by growth assays on plates containing low concentrations of cytotoxic peptides that bind to endogenous phosphatidylserine (papuamide B) and phosphatidylethanolamine (duramycin) [25]. When a RGSH10:yeALA1 fusion was expressed alone in the *Δdrs2Δdnf1Δdnf2* strain under the control of a galactose-inducible promoter, the plant P4 ATPase failed both to complement the cold-sensitive phenotype and to restore transport of endogenous phosphatidylserine or phosphatidyletanolamine (Figure S3). Plasma membrane localized P4 ATPases which have phosphatidylcholine as a substrate are in some cases capable of transporting the toxic choline ether lipid miltefosine to the interior of the cell [17]. Employing this assay, we were unable to demonstrate yeALA1-mediated internalization of miltefosine (Figure S3). Identical results were obtained for an untagged yeALA1.

A possible explanation for the apparent inability of *ALA1* to complement the phenotypes of the *Δdrs2Δdnf1Δdnf2* yeast mutant strain could be a defect in targeting of the plant protein expressed in the yeast cells. In order to investigate whether lack of complementation could be due to protein mistargeting in the heterologous host, plasma membrane-enriched membrane fractions from yeast expressing RGSH10-tagged ALA1 alone or together with a representative member of the ALIS family (RGSH6:ALIS1) were subjected to discontinuous sucrose gradient fractionation. The results indicate that ALA1 is in fact capable of reaching the plasma membrane when overexpressed in yeast, both alone and with ALIS1 (Figure S4). Similar results have been reported previously for other members of the ALA family [25], [26].

## Discussion

In this work we provide the first evidence for the presence of a P4 ATPase in plant plasma membranes. In animal cells, P4 ATPases have been implicated in generation of phospholipid asymmetry across the two bilayers of the membrane, a process hypothesized to be a prerequisite for endocytosis and other important functions of the plasma membrane. Expression of fluorescently-tagged versions of ALA1 in leaves of *Nicotiana benthamiana* proved that the protein is retained in the ER when no putative β-subunit is present. Co-expression with each of three different Arabidopsis ALIS proteins (ALIS1, ALIS3 and ALIS5) resulted in ALA1 exit from the ER and trafficking of this protein to the plasma membrane, demonstrating that this P4 ATPase is also strictly dependent on interaction with a β-subunit to reach its final subcellular localization. Moreover, when YFP-tagged versions of the ALIS proteins (normally retained in the ER; [25]) were co-expressed with GFP-tagged ALA1, both fluorescent signals could be co-localized in the plasma membrane, indicating that both proteins travel together to the destination membrane. These results are in agreement with previous studies with Golgi-localized ALA3 and prevacuolar compartment-localized ALA2, which indicate that the localization determinants for the complex formed between the P4 ATPase and its β-subunit are present in the α -catalytic subunit [25].

We further aimed at expressing ALA1 in a *Δdrs2Δdnf1Δdnf2* yeast strain, which has successfully been used before for characterization of lipid translocation by the plant P4 ATPases ALA2 and ALA3 [25], [26]. After optimization of the expression levels for ALA1 in yeast, we were successful in expressing recombinant protein in amounts that could readily be detected in yeast extracts by immunological methods. Following expression of *ALA1* alone and together with *ALIS* genes we were not able to demonstrate functional complementation of the cold-sensitive Drs2p-related phenotype or to recover the loss of phospholipid asymmetry generated by deletion of the endogenous P4 ATPases in the mutant yeast strain. This lack of complementation was not derived from a defect in targeting of ALA1 within the yeast trafficking machinery, as the plant P4 ATPase overexpressed in yeast was found to reach the yeast plasma membrane both in the presence or the absence of an ALIS β-subunit. We could not detect any ATPase activity above background levels in membranes from transformed yeast cells and also failed to demonstrate the formation of an acyl-phosphate bond in the recombinant protein (data not shown). The inability of ALA1 expression to complement the phenotypes of the mutant yeast strain could therefore be due to lack of functionality of the recombinant protein, for example if an unknown factor (*e.g.* protein or lipid) present in plants but not in yeast is required for ALA1 activity.

Previously, Gomez et al. [27] demonstrated that *ALA1* complements the cold-sensitive phenotype of the *Δdrs2* mutant strain [30] in the absence of a co-expressed ALIS subunit. We made several unsuccessful attempts to reproduce the complementation in the same *Δdrs2* mutant strain, in order to rule out discrepancies due to the yeast background. This could imply that ALA1 is not a functional homologue of yeast Drs2p. However, we noted that this strain gives rise to an elevated number of phenotypic revertants, especially when incubated at low temperatures. Revertants could arise from induction in yeast of factors that facilitate function of recombinant ALA1. Several yeast P4 ATPases have been shown to require interaction with other proteins for full activity. For instance, plasma membrane-localized Dnf1p and Dnf2p are known to require phosphorylation by the kinases Fpk1p and Fpk2p in order to be active at their final location [31]. Another example is the interaction of Drs2p with ARF-GEF, a small GTPase involved in vesicle production in the TGN. This protein-protein interaction significantly stimulates lipid translocation by Drs2p [32]. Furthermore, Drs2p activity is dependent on the presence of a specific regulatory lipid [33]. Lack of complementation by ALA1 of the yeast P4 ATPase deletion mutant phenotypes could therefore be due to lack of an unknown factor required for protein activity, e.g. a regulatory lipid, an activating kinase and/or other interacting proteins.

In conclusion, we have shown that ALA1 localizes to the plasma membrane in plant cells and have a strict requirement for a β-subunit to exit the ER and reach its final location.

## Materials and Methods

### *In silico* Analysis of DNA Sequences

Primers were designed on the basis of information contained in the P-type ATPase database (http://traplabs.dk/patbase). The genomic DNA sequence for the ALA1 gene and its adjacent 5′UTRs was extracted from The Arabidopsis Information Resource webpage (www.arabidopsis.org). Transcription termination signal predictions were performed online at http://harlequin.jax.org/drupal/?q=yeastRNApred [34]. A graphical codon usage analyzer (http://gcua.schoedl.de/) was used to identify rare codons that might result in low levels of expression of ALA1 in *S. cerevisiae*. Default parameters were selected in both cases. Two different *ALA1* modified versions were designed based in these analyses, *ALA1noTT* (no transcription termination) and *yeALA1* (yeast-enhanced). For *ALA1noTT*, a predicted transcription termination signal sequence around 2800 bp is removed, while *yeALA1* lacks this signal and contains five silent mutations that modify the codon usage for arginine from the plant CGG to the yeast-favoured AGA and AGG. Figure S2 shows a comparison of *ALA1* to the mutated versions of the gene.

### DNA Cloning

All Polymerase Chain Reactions (PCRs) carried out in this study were performed using Phusion High-Fidelity DNA polymerase (Finnzymes) under the standard conditions recommended by the manufacturer. In the case of genomic DNA amplification, a 2% final concentration of dimethyl sulfoxide (DMSO) was added to the reaction mixture. A full list of the primers used and their characteristics can be found in Table S1. Table S2 comprises a list of the plasmids obtained by cloning PCR products into commercially available blunt-ended vectors, including a summary of the primers and templates used for amplification. In some cases, an overlapping PCR strategy was used to introduce modifications in the original gene sequence (e.g. eliminate a predicted transcription termination site) or to generate gene fusions (e.g. *ALA1::GFP* under the control of the *ALA1* promoter). In these cases, a first round of PCR amplifications was carried out to obtain overlapping fragments containing the desired modifications. In a second round, 1–2 µL of each of these PCR reactions were mixed together and used as template with primers that amplify the full-length product. The pENTR™/D-TOPO® Cloning Kit and the Zero Blunt*®* TOPO*® PCR* Cloning Kit for Sequencing were used for introducing the PCR fragments into the Gateway®-compatible vector pENTR™/D-TOPO® (Invitrogen) and pCR4®Blunt-TOPO® vector (Invitrogen), respectively. All PCR products were fully sequenced in these plasmids before further cloning. Tables S3 and S4 contain a summary of all yeast and plant plasmids, respectively, generated in this work.

For expression in yeast, the cDNA sequence corresponding to ALA1 (At5g04930) was PCR amplified both in untagged and hemaglutinin (HA) epitope-tagged forms and cloned into pENTR™/D-TOPO®. Gateway® technology was then used to transfer the ALA1-codifying sequences to a Gateway®-compatible version of yeast plasmid pRS423-GAL [35] and its derivatives containing *RGSH6:ALIS* gene fusions [26]. These plasmids bear a bi-directional *GAL1-10* promoter fusion that allows for coordinated expression of two genes, as well as a HIS3 cassette for selection of yeast transformants in media lacking histidine. A modified version of *ALA1* lacking a predicted transcription termination site, *ALA1noTT* (see above and Figure S2), was generated by overlapping PCR and cloned into yeast plasmids as above.

In order to increase protein expression from the *GAL1-10* promoter in pRS423-GAL [35], we modified this promoter to include the leader sequence of the yeast *PMA1* gene. Pma1p is a yeast plasma membrane H^+^-ATPase, which can represent up to 15% of the total protein at this membrane [36]. This high level of expression is dependent on the strong constitutive *PMA1* promoter. A piece of 238 bp of this *PMA1* promoter from the transcription start point to the start codon [37] was fused to the *GAL10* side of the *GAL1-10* promoter in plasmid pRS423-GAL using an overlapping PCR strategy (see Tables S1 and S2 for details). A RGSH10 epitope and a Tobacco Etch Virus (TEV) protease cleavage site separated by a 3-glycine linker were engineered at the end of the *PMA1* leader sequence in order to allow for immunological detection of expressed proteins. The PCR product cloned in pCR4®Blunt-TOPO® (Invitrogen) was digested with restriction enzymes *Bam*HI and *Eco*RI and transferred to pRS423-GAL treated with the same enzymes, generating plasmid pMP4062. A version of this plasmid not containing the epitope tag (pMP4075) was generated by PCR amplification of the promoter-leader fusion with unmodified primers (see Tables S1 and S2 for details) and cloning as above.

For further optimization of ALA1 expression in yeast, another modified version of the gene, *yeALA1* (for a description see above and Figure S2), was produced by overlapping PCR and cloned into the pCR4®Blunt-TOPO® vector. After full sequencing, *yeALA1* was transferred to yeast plasmids pMP4062 and pMP4075 using restriction enzymes *Eco*RI and *Sal*I. In order to introduce other versions of the *ALA1* gene in these modified yeast plasmids, the clones containing *ALA1* and *ALA1noTT* in the pRS423-GAL backbone (pMP3560 and pMP3647, respectively, see Table S3) were digested with *Age*I and *Blp*I. After DNA electrophoresis on agarose gels, the approximately 9 kb long fragment was isolated using a Gel Advanced™ Gel Extraction System (Viogene, Taiwan). This eliminates part of the promoter and a fragment of around 170 bp of the *ALA1* gene. The linearized plasmids were transformed into a wild type yeast strain (see below) together with a PCR fragment containing the full length *GAL1-10* promoter::*PMA1* leader fusion and the first 250 nt of the *ALA1* gene both in tagged and untagged versions (see Tables S1 and S2 for details on the PCR strategy). After homologous recombination, plasmid DNA was extracted from the yeast cells by heating at 95°C for 4 min in the presence of 0.2% sodium dodecyl sulfate (SDS). After removal of cellular debris by centrifugation, 1 µL of the supernatant was used for electroporation of *E. coli* cells. Amplified plasmids were tested by restriction and sequenced to ensure the integrity of the DNA construct. Further yeast transformations for functional characterization (see below) were carried out with these sequenced plasmids.

Introduction of *ALA1* and *ALA1noTT* (both in HA-tagged and untagged versions) into yeast plasmids already containing RGSH6-tagged versions of *ALIS* (see above) had proven to be a tedious task and mutations in both the *ALA1* and the *ALIS* gene sequences were often occurring during the cloning process, suggesting a toxic effect of these constructs for *E. coli* cells. In order to ease the cloning process, a two-plasmid transformation in yeast was used for co-expression of new *ALA1* constructs with *ALIS* genes. For this purpose, plasmids containing *RGSH6:ALIS* in the pRS423-GAL backbone [26] were digested with *Bam*HI and *Sac*I and the 1-kb fragments corresponding to the tagged *ALIS* were ligated into pRS426-GAL [35] treated with the same enzymes. This plasmid contains the bi-directional *GAL1-10* promoter and a URA3 cassette for selection of transformants on media lacking uracil.

For expression of ALA1 *in planta*, the genomic DNA fragments corresponding to *ALA1* and 2 kb of its adjacent 5′UTR sequences were cloned by PCR from a total genomic DNA preparation (see Tables S1 and S2 for details). After cloning into pCR4®Blunt-TOPO® vector as described above, the new plasmids were used as templates in an overlapping PCR to generate an *ALA1* promoter::*ALA1* genomic DNA::GFP fusion, that bears artificial restriction sites *Xho*I and *Bst*EI at the 5′- and 3′-ends, respectively. The DNA fusion digested with *Xho*I and *Bst*EI was cloned into pCAMBIA1302 (CAMBIA, Brisbane, Australia) digested with *Sal*I and *Bst*EI. To clone *ALA1* under the control of a double 35 S promoter, which should increase expression levels, new PCR amplifications were carried out to clone the genomic DNA fragment into vector pENTR™/D-TOPO® as described above. Two individual PCR products containing or lacking a stop codon were obtained. Gateway® technology was used to further clone these products into pMDC43 or pMDC83 [28] to generate N- or C-terminal end fusions of GFP to ALA1.

### Yeast Strains and Media

Wild type *S. cerevisiae* strain w303-1a (*Mat* a *ade2-1 can1-100 his3-11,15 leu3,112 trp1-1 ura3-1*) was used for cloning different versions of *ALA1* into yeast plasmids by homologous recombination (see above). Functional complementation was carried out employing *S. cerevisiae* mutant strain ZHY709 (*MATα his3 leu2 ura3 met15 dnf1Δ dnf2Δ drs2::LEU2*; [38]). Cells were grown at 30°C in standard rich medium with glucose (YPD) or galactose (YPG), or selective SD or SG media [39] containing Yeast Synthetic Drop-out Medium Supplement without the required amino acids (Sigma). Solid media were added 2% agar [40]. Papuamide B (Flintbox, Lynsey Huxham), duramycin (Sigma-Aldrich), and miltefosine (Calbiochem) were added to rich synthetic SD or SG media to final concentrations 0.05 µg/mL, 1.5 µM and 2.5 µg/mL, respectively.

### Yeast Transformation and Growth

Yeast cells were transformed by the lithium acetate method [41]. Transformants were incubated in liquid SG medium for 4 h and then diluted with water to OD_600_ 0.05 and 0.005. Drops (5 µL) were spotted on plates and incubated at 20°C for 6–8 days (cold) or at 30°C for 2–3 days. All experiments were repeated independently at least three times. For isolation of total membrane protein fractions for immunodetection, 30–40 yeast colonies transformed with the desired plasmid(s) were inoculated in 100 mL of liquid SG medium in 250 mL flasks and grown at 30°C with 140 rpm shaking. After 24 hurs growth, cells were inoculated in 1 L of fresh SG medium in 2 L flasks and allowed to grow under the same conditions for a further 24 hours.

### Yeast Membrane Preparation and Protein Immunodetection

Isolation of total cellular yeast membranes for protein expression analysis was performed as previously described [40]. Discontinuous sucrose gradient fractionation of plasma membrane-enriched membrane fractions was carried out essentially as described in [25] except that cells were grown for 24 hours (28^o^C, 160 rpm) in 50 mL selective SG media and then inoculated without washing into 500 mL fresh selective SG media before incubation for further 16 hoursF under the same conditions prior to membrane preparation. Protein samples were quantified by the method of Bradford using bovine serum albumin (BSA) as a standard. For protein blot analysis of membrane fractions, 30 µg total protein were precipitated with trichloroacetic acid (TCA), loaded onto SDS-PAGE gels, transferred to nitrocellulose membranes and immunodetected as previously described [26]. Detection of HA- and histidine (RGSH6 or RGSH10)-tagged proteins was carried out using a monoclonal anti-HA antibody (Sigma) and an anti-RGSHis antibody BSA-free (Qiagen), respectively. Dpm1p was immunodetected using an anti-dolichol phosphate mannose synthase antibody (Molecular Probes), Sed5p with affinity-purified anti-Sed5p [42] and Pma1p with a polyclonal antibody raised against its C terminus [43]. Bands were visualized with the 5-bromo-4-chloro-3-indolyl phosphate/nitroblue tetrazolium (BCIP/NBT) color development substrate (Promega).

### Transient Expression in Tobacco Epidermal Leaf Cells

*Agrobacterium tumefaciens* strain C58C1 [44] was transformed by electroporation and transformants selected on YEP plates (1% yeast extract, 2% peptone, 1.5% agar) containing 25 µg/mL gentamycin and 50 µg/mL kanamycin. Transient expression in tobacco epidermal cells was carried out as described [25] using three week old *N. benthamiana* plants. Expression was visualized 4–5 days after infiltration. Plasmolysis of epidermal tobacco cells was achieved by infiltrating the leaves with a 1 M mannitol solution 1–2 min prior to sample preparation for microscopic visualization.

### Confocal Microscopy

A Leica TCS SP2/MP or SP5 II spectral confocal laser scanning microscope (Leica Microsystems, Heidelberg, Germany) with a 63x/1.2 numerical aperture water immersion objective were used as previously described [26]. Both GFP and YFP were excited at 488 nm and emission spectra were recorded between 495 and 510 nm for GFP (green channel) and 530 and 545 nm for YFP (magenta channel). Sequential scanning between lines was used to follow both fluorescent proteins at once. Autofluorescence was visualized between 600 and 680 nm. No fluorescence bleed-through of YFP or GFP signals was detected under our experimental conditions (see Figure S1).

## Supporting Information

Figure S1 **Bleed-through control of cells expressing GFP- and YFP-tagged proteins.** A *GFP::ALA1* fusion was transiently expressed in tobacco epidermal cells in the presence of an YFP tagged ALIS3. The image shows plasmolyzed cells subjected to osmotic shock in the presence of a concentrated mannitol solution. (**A**) *Green:* GFP fluorescence; (**B**) *magenta:* YFP fluorescence; (**C**) *gray:* bright field image. (**D**) overlay image. Two adjacent cells containing only GFP or only YFP signals are shown. In the upper part of the image, a cell containing both signals can be seen. Scale bar: 25 µm.
(TIF)

Figure S2 **Alignment showing a comparison between** ***ALA1*** **cDNA and the modified version used in for increasing expression of the protein in yeast.** The unmodified *ALA1* cDNA is highlighted in yellow. Silent mutations are highlighted in red (modification of 5 codons codifying for arginine and deletion of restriction sites) or magenta (elimination of a putative transcription termination signal).
(TIF)

Figure S3 ***ALA1*** **fails to complement the phenotypes of a yeast** ***Δdrs2Δdnf1Δdnf2*** **mutant strain.** A version of the *ALA1* cDNA modified to eliminate a putative transcription termination site and five rare codons codifying for Arg (*yeALA1*) was expressed alone or together with an ALIS in yeast lacking the three endogenous P4 ATPases Drs2p, Dnf1p and Dnf2p. *yeALA1* was expressed untagged or was tagged at the N-terminal end with an RGSH10-tag while ALIS proteins bear a RGSH6-tag at the same end. Left to right: *glucose*: control showing uninduced cells; *galactose:* control plate grown under standard conditions; *20°C*: cells grown on galactose at the restrictive growth temperature; *duramycin*: cells grown on galactose plates containing 1.5 µM of the phosphatidylethanolamine-binding cytotoxic peptide duramycin; *Pap. B*: cells grown on galactose plates containing 0.05 µL/mL of the phosphatidylserine-binding cytotoxic peptide papuamide B; *miltefosine*: cells grown on galactose plates containing 2.5 µL/mL of the cytotoxic choline ether lipid miltefosine.
(TIF)

Figure S4 **ALA1 reaches the plasma membrane when expressed in yeast.** PM-enriched membranes from yeast expressing *RGSH10:ALA1* alone or *RGSH10:ALA1* together with *RGSH6:ALIS1* were subjected to discontinuous sucrose density gradient fractionation. Fractions corresponding to 30 and 48% sucrose, enriched respectively in endomembranes (ER, Golgi) and plasma membranes, were analyzed. Western blots were probed using the following antibodies: anti-Pma1p, plasma membrane; anti-Sed5p, Golgi apparatus; anti-Dpm1p, ER; and anti-RGSHis, ALA1 and ALIS1.
(TIF)

Table S1 **PCR Primers used in this work.** Standard PCR was used to amplify the ALA1 genomic and cDNA fragments and the ALA1 promoter region. Overlapping PCR strategies were designed to modify the ALA1 cDNA fragment and the plasmid used for overexpression in yeast, as well as to generate a fusion of the ALA1 genomic fragment to its natural promoter for *in planta* expression. See Materials and Methods for further details. Artificial restriction sites before the ATG start codon or after the stop codon are marked in bold; Epitope tags are mark in bold and italics; New restriction sites generated by silent mutation within the codifying sequence are written in italics; Restriction sites (in brackets) and transcription termination sites eliminated by silent mutations are written in italic lowercase letters; Silent mutations that generate modifications of the codon usage are written in lowercase letters; The CACC sequence included at the beginning of some oligos is a requirement for cloning in the Gateway®-compatible vector pENTR™/D-TOPO®.
(DOC)

Table S2 **Plasmids generated by cloning of PCR products into commercial blunt-ended plasmids.** ALA1 no TT: modified ALA1 gene in which a predicted transcription termination signal has been deleted; yeALA1: modified ALA1 gene in which a predicted transcription termination signal has been deleted and several codons codifying for arginine have been substituted to match the yeast preferred codon usage (see Materials and Methods and Figure S2). In some cases, the final PCR products have been generated by overlapping PCR. In these cases, the subsequent PCR amplification rounds are named PCR1 (gegneration of overlapping fragments) and PCR2 (amplification of the full-length final product). Letters A to E in PCR1 refer to the different fragments generated in individual PCR reactions during the first amplification round.
(DOC)

Table S3 **Plasmids generated for expression of ALA1 and ALIS proteins in** ***S. cerevisiae*** **.** ALA1 no TT: modified ALA1 gene in which a predicted transcription termination signal has been deleted; yeALA1: modified ALA1 gene in which a predicted transcription termination signal has been deleted and several codons codifying for arginine have been substituted to match the yeast preferred codon usage (see Materials and Methods and Figure S2).
(DOC)

Table S4 **Plasmids for expression of fluorescently-tagged ALA1** ***in planta.***
(DOC)
