# Supplementary figures and images for: A Putative Plant Aminophospholipid Flippase, the Arabidopsis P4 ATPase ALA1, Localizes to the Plasma Membrane following Association with a β-Subunit
Source: PLoS One. 2012 Apr 13;7(4):e33042. doi: 10.1371/journal.pone.0033042 (PMC3326016; doi:10.1371/journal.pone.0033042)

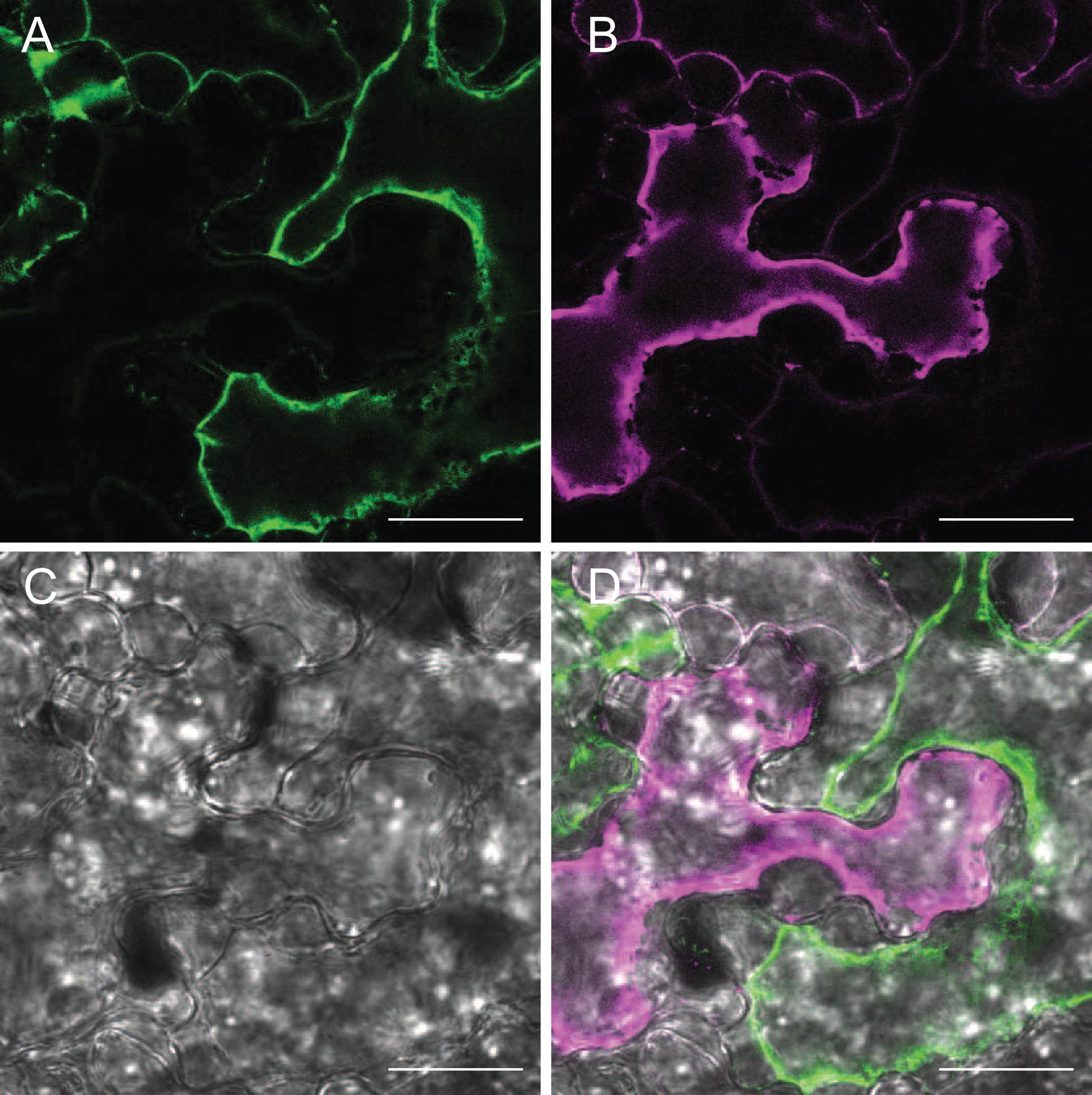

Supplement: Figure S1 — Bleed-through control of cells expressing GFP- and YFP-tagged proteins. A GFP::ALA1 fusion was transiently expressed in tobacco epidermal cells in the presence of an YFP tagged ALIS3. The image shows plasmolyzed cells subjected to osmotic shock in the presence of a concentrated mannitol solution. (A) Green: GFP fluorescence; (B) magenta: YFP fluorescence; (C) gray: bright field image. (D) overlay image. Two adjacent cells containing only GFP or only YFP signals are shown. In the upper part of the image, a cell containing both signals can be seen. Scale bar: 25 µm. (TIF) [file pone.0033042.s001.tif]

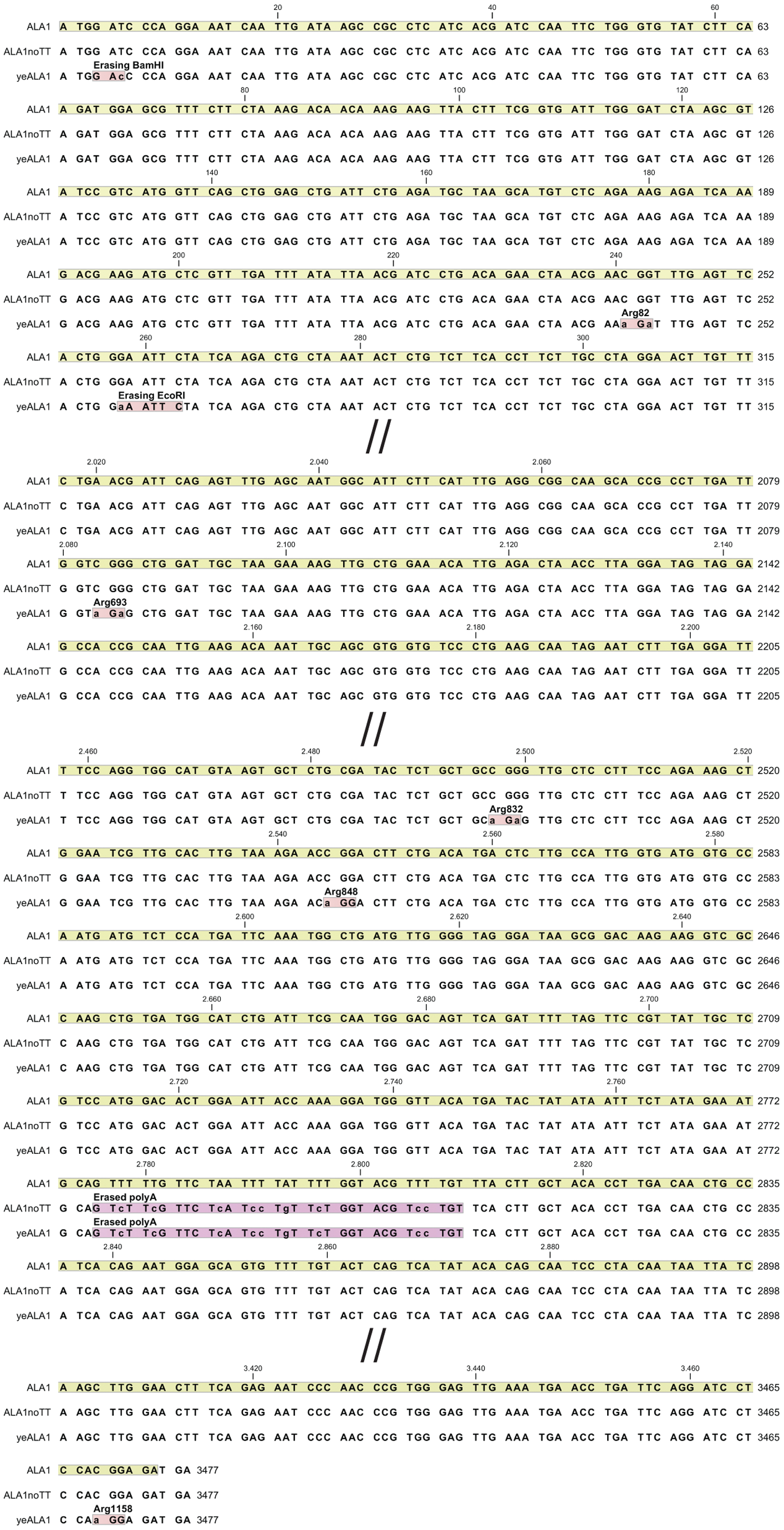

Supplement: Figure S2 — Alignment showing a comparison between ALA1 cDNA and the modified version used in for increasing expression of the protein in yeast. The unmodified ALA1 cDNA is highlighted in yellow. Silent mutations are highlighted in red (modification of 5 codons codifying for arginine and deletion of restriction sites) or magenta (elimination of a putative transcription termination signal). (TIF) [file pone.0033042.s002.tif]

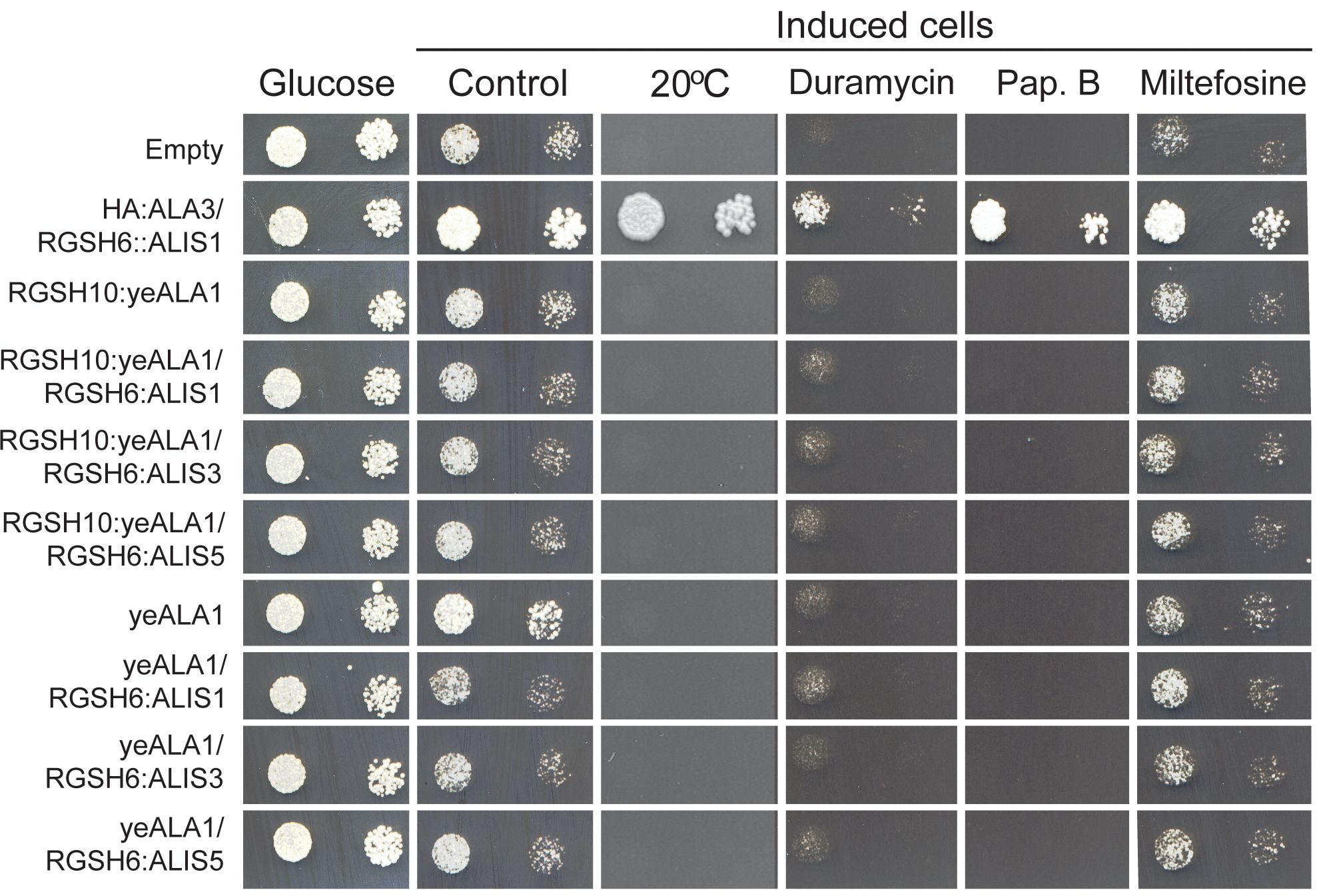

Supplement: Figure S3 — ALA1 fails to complement the phenotypes of a yeast Δdrs2Δdnf1Δdnf2 mutant strain. A version of the ALA1 cDNA modified to eliminate a putative transcription termination site and five rare codons codifying for Arg (yeALA1) was expressed alone or together with an ALIS in yeast lacking the three endogenous P4 ATPases Drs2p, Dnf1p and Dnf2p. yeALA1 was expressed untagged or was tagged at the N-terminal end with an RGSH10-tag while ALIS proteins bear a RGSH6-tag at the same end. Left to right: glucose: control showing uninduced cells; galactose: control plate grown under standard conditions; 20°C: cells grown on galactose at the restrictive growth temperature; duramycin: cells grown on galactose plates containing 1.5 µM of the phosphatidylethanolamine-binding cytotoxic peptide duramycin; Pap. B: cells grown on galactose plates containing 0.05 µL/mL of the phosphatidylserine-binding cytotoxic peptide papuamide B; miltefosine: cells grown on galactose plates containing 2.5 µL/mL of the cytotoxic choline ether lipid miltefosine. (TIF) [file pone.0033042.s003.tif]

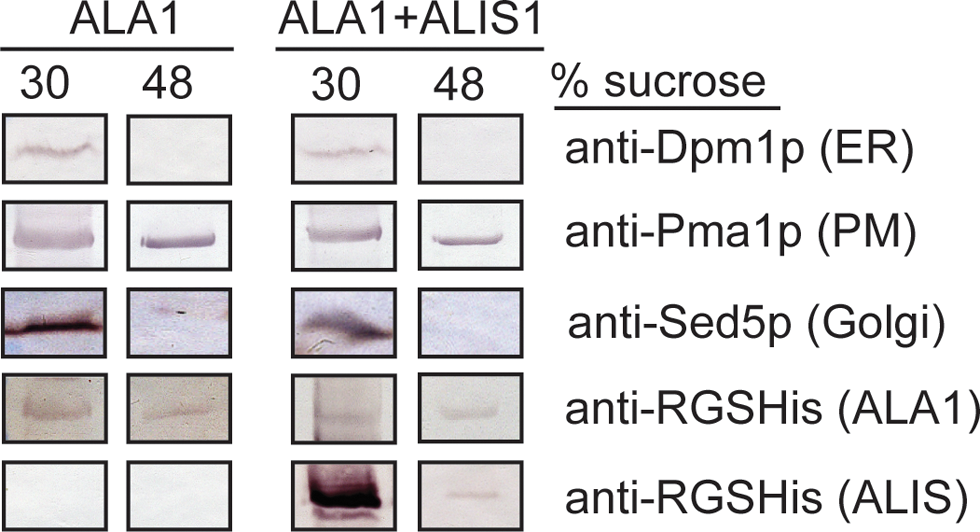

Supplement: Figure S4 — ALA1 reaches the plasma membrane when expressed in yeast. PM-enriched membranes from yeast expressing RGSH10:ALA1 alone or RGSH10:ALA1 together with RGSH6:ALIS1 were subjected to discontinuous sucrose density gradient fractionation. Fractions corresponding to 30 and 48% sucrose, enriched respectively in endomembranes (ER, Golgi) and plasma membranes, were analyzed. Western blots were probed using the following antibodies: anti-Pma1p, plasma membrane; anti-Sed5p, Golgi apparatus; anti-Dpm1p, ER; and anti-RGSHis, ALA1 and ALIS1. (TIF) [file pone.0033042.s004.tif]
