# Supplementary material for: A Putative Plant Aminophospholipid Flippase, the Arabidopsis P4 ATPase ALA1, Localizes to the Plasma Membrane following Association with a β-Subunit
Source: PLoS One. 2012 Apr 13;7(4):e33042. doi: 10.1371/journal.pone.0033042 (PMC3326016; doi:10.1371/journal.pone.0033042)
Supplement: Table S1 — PCR Primers used in this work. Standard PCR was used to amplify the ALA1 genomic and cDNA fragments and the ALA1 promoter region. Overlapping PCR strategies were designed to modify the ALA1 cDNA fragment and the plasmid used for overexpression in yeast, as well as to generate a fusion of the ALA1 genomic fragment to its natural promoter for in planta expression. See Materials and Methods for further details. Artificial restriction sites before the ATG start codon or after the stop codon are marked in bold; Epitope tags are mark in bold and italics; New restriction sites generated by silent mutation within the codifying sequence are written in italics; Restriction sites (in brackets) and transcription termination sites eliminated by silent mutations are written in italic lowercase letters; Silent mutations that generate modifications of the codon usage are written in lowercase letters; The CACC sequence included at the beginning of some oligos is a requirement for cloning in the Gateway®-compatible vector pENTR™/D-TOPO®. (DOC) [file pone.0033042.s005.doc]

| Primers for ALA1 cDNA cloning and gene optimization for yeast expression | | |
| --- | --- | --- |
| Primer name | Sequence 5’-3’ | Modifications |
| oli1966B | CACC**CACGGAGTG**ATGGATCCCAGGAAATC | **DraIII** |
| oli2096 | **GTCGAC**CTATCT*CCGCGG*AGGATCCTGAATCAGG | **SalI**, *SacII* |
| oli1967B | **GTCGAC**TCT*CCGCGG*AGGATCCTGAATC | **SalI**, *SacI,*no STOP codon |
| oli2389 | ATT**CACGGAGTG*ATGTACCCATACGATGTTCCAGATTACGCT***GATCCCAGGAAATCAATTGATAAG CC | **DraIII**, HA |
| oli2467 | CACCATGTACCCATACGATGTTCC | Introduction of A at position -3 with respect to ATG on HA- tagged ALA1 |
| oli3178 | CACCAAAATGGATCCCA GGAAATCAAT TG | Introduction of A at position -3 with respect to ATG in untagged ALA1 |
| oli3175 | CTATAGAAATGCAGT*cTTcGTTCTcATccTgTTcTGGTACGTcc*TGTTCTGGTACGTCCTGTTCACTTGCTACACCTTGACAACTGCC | Elimination of transciption termination signal around 2800bp (Forward) |
| oli3176 | GGCAGTTGTCAAGGTGTAGCAAGTGAACA*ggACGTACCAgAAcAggATgAGAACg*AAgACTGCATTTCTATAG | Elimination of transciption termination signal around 2800bp (Reverse) |
| oli3304 | **GAATTC**ATCAT*ggaccc*CAGGAAATCAATTG | **EcoRI**, (*BamHI*) |
| oli3305 | CTAACGAAaGaTTTGAGTTCACTGG*aaattc*TATCAAGAC | (*EcoRI*), modified codon for Arg82 (Forward) |
| oli3306 | GTCTTGATA*gaattt*CCAGTGAACTCAAAtCtTTCGTTAG | (*EcoRI*), modified codon for Arg82  (Reverse) |
| oli3309 | GATTGGTaGaGCTGGATTGC | modified codon for Arg693 Forward |
| oli3310 | GCAATCCAGCtCtACCAATC | modified codon for Arg693 Reverse |
| oli3311 | CTGCaGaGTTGCTCCTTTCCAGAAAGCTGGAATCGTTGCACTTGTAAAGAACaGGACTTC | modified codons for Arg832 and Arg848  Forward |
| oli3312 | GAAGTCCtGTTCTTTACAAGTGCAACGATTCCAGCTTTCTGGAAAGGAGCAACtCtGCAG | modified codon for Arg832 and Arg848  Reverse |
| oli3313 | **GTCGAC**TCATCTCCtTGGAGGATCCTG | SalI, modified codon for Arg1158 |
| Primers for generation of expression-optimized yeast promoters | | |
| Primer name | Sequence 5’-3’ ^a^ | Purpose |
| oli3424 | CTCAGCTTTGCTAAAGTGCAAAAAGTCG | Amplification of 1kb of pma1 UTRs- Forward |
| oli3425 | ATTGATATTGTTTGATAATTAAATCTTTC | Amplification of 1kb of pma1 5’UTRs- Reverse |
| oli3373 | GATATAGAAGAAAAAAAGATTTTCACTATTGGTGAATTTTCAAAAATTCTTACTTTTTTTTTGGATGG | Overlap pma1 leader - GAL10 promoter Forward |
| oli3374 | CCATCCAAAAAAAAAGTAAGAATTTTTGAAAATTCACCAATAGTGAAAATCTTTTTTTCTTCTATATC | Overlap pma1 leader – GAL10 promoter Reverse |
| oli3375 | **GGATCC**tcttGTCGTCATCGTCTTTGTAGTCTGCCATtataGTTTTTTCTCCTTGACG | Optimization of GAL1 promoter sequence/amplification of full-length GAL1-10 promoter Forward |
| oli3376 | ***ACCACCTTGAAAATACAAATTTTCACCACCACCATGATGATGATGATGATGATGATGATGATGAGAACCTCTAGCCAT***ATTGATATTGTTTGATAATTAAATCTTTCTTATCTTCTTATT | Including tag after pma1 leader sequence (RGSH10-G3-TEV cleavage site-G2) |
| Cloning of ALA1 promoter and genomic DNA sequence for expression in planta | | |
| Primer name | Sequence 5’-3’ ^b^ | Purpose |
| oli3347 | **CTCGAG**AATATTAAAACCAATACTCGATCGATAATGACCAATTTG | Amplification of 2kb of ALA1 5’UTRs, **XhoI**- Forward |
| oli3348 | TTCCCGGGTCCAGGAATTGGATTTGGACAATG | Amplification of 2kb of ALA1 5’UTRs- Reverse |
| oli3349 | ATGGATCCCAGGAAATCAATTGATAAGCC | Amplification of the genomic piece of DNA coding for ALA1-Forward |
| oli3350 | **GGTGACC**TCATCTCCGTGGAGGATCCTGAATCAGGTTCATTTC | Amplification of the genomic piece of DNA coding for ALA1, **BstEII**-Reverse |
| oli3351 | **GGTGACC**TCACACGTGGTGGTGGTGGTGG | Amplification of mGFP5, **BstEII** - Reverse |
| oli3352 | GTCCAAATCCAATTCCTGGACCCGGGAAATGGATCCCAGGAAATCAATTGATAAGCC | overlap ALA1promoter-ALA1 genomic DNA- Forward |
| oli3353 | GGCTTATCAATTGATTTCCTGGGATCCATTTCCCGGGTCCAGGAATTGGATTTGGAC | overlap ALA1promoter-ALA1 genomic DNA-Reverse |
| oli3354 | TGAACCTGATTCAGGATCCTCCACGGAGAATGGTAGATCTGACTAGTAAAGGAGAAGAAC | overlap ALA1 genomic DNA-GFP Forward |
| oli3355 | GTTCTTCTCCTTTACTAGTCAGATCTACCATTCTCCGTGGAGGATCCTGAATCAGGTTCA | overlap ALA1 genomic DNA-GFP- Reverse |

^a^In overlapping primers, underlined nucleotides correspond to the PMA1 leader sequence

^b^In overlapping primers, underlined nucleotides correspond to the ALA1 sequence
