# Supplementary material for: A Putative Plant Aminophospholipid Flippase, the Arabidopsis P4 ATPase ALA1, Localizes to the Plasma Membrane following Association with a β-Subunit
Source: PLoS One. 2012 Apr 13;7(4):e33042. doi: 10.1371/journal.pone.0033042 (PMC3326016; doi:10.1371/journal.pone.0033042)
Supplement: Table S2 — Plasmids generated by cloning of PCR products into commercial blunt-ended plasmids. ALA1 no TT: modified ALA1 gene in which a predicted transcription termination signal has been deleted; yeALA1: modified ALA1 gene in which a predicted transcription termination signal has been deleted and several codons codifying for arginine have been substituted to match the yeast preferred codon usage (see Materials and Methods and Figure S2). In some cases, the final PCR products have been generated by overlapping PCR. In these cases, the subsequent PCR amplification rounds are named PCR1 (gegneration of overlapping fragments) and PCR2 (amplification of the full-length final product). Letters A to E in PCR1 refer to the different fragments generated in individual PCR reactions during the first amplification round. (DOC) [file pone.0033042.s006.doc]

| Cloning in blunt-ended PCR compatible commercial vectors | | | | |
| --- | --- | --- | --- | --- |
| Plasmid name | Insert | Backbone | Template | Primers used |
| pMP2016 | ALA1 | pENTR™/D-TOPO® | Total cDNA | oli1966B/oli2096 |
| pMP2430 | HA::ALA1 | pENTR™/D-TOPO® | pMP2016 | oli2389/oli2096 |
| pMP3559 | (-3A)HA::ALA1 | pENTR™/D-TOPO® | pMP2430 | oli2467/oli2096 |
| pMP3595 | ALA1 no TT | pENTR™/D-TOPO® | PCR1: pMP2016 | oli3178/oli3176 (A) |
|  |  |  |  | oli3175/oli2096 (B) |
|  |  |  | PCR2: Fragments A and B from PCR1 | oli3178/oli2096 |
| pMP3598 | HA::ALA1 no TT | pENTR™/D-TOPO® | PCR1:pMP3559 | oli2476/oli3176 (A) |
|  |  |  |  | oli3175/oli2096 (B) |
|  |  |  | PCR2: Fragments A and B from PCR1 | oli2476/oli2096 |
| pMP3716 | yeALA1 | pCR4®Blunt-TOPO® | PCR1: pMP3595 | oli3304/oli3306 (A) |
|  |  |  |  | oli3305/oli3308 (B) |
|  |  |  |  | oli3307/oli3310 (C) |
|  |  |  |  | oli3309/oli3312 (D) |
|  |  |  |  | oli3311/oli3313 (E) |
|  |  |  | PCR2: Fragments A-E from PCR1 | oli3304/oli3313 |
| pMP4081 | PMA1 5’UTRs | pCR4®Blunt-TOPO® | Total yeast genomic DNA | oli3424/oli3425 |
| pMP4082 | GAL1-10 prom.::PMA1 leader::RGSH10-TEV | pCR4®Blunt-TOPO® | PCR1: pMP4081 (A) | oli3376/oli3373 (A) |
|  |  |  | pMP1965^a^ (B) | oli3374/oli3375 (B) |
|  |  |  | PCR2: Fragments A and B from PCR1 | oli3376/oli3375 |
| pMP4080 | GAL1-10 prom.::PMA1 leader | pCR4®Blunt-TOPO® | pMP4082 | oli3425/oli3375 |
| pMP3930 | ALA1 genomic DNA | pCR4®Blunt-TOPO® | Total Arabidopsis genomic DNA | oli3340/oli3350 |
| pMP3931 | ALA1 5’UTRs (2kb) | pCR4®Blunt-TOPO® | Total Arabidopsis genomic DNA | oli3347/oli3348 |
| pMP3932 | ALA1 5’UTRs::ALA1 genomic DNA::mGFP5 | pCR4®Blunt-TOPO® | PCR1: pMP3931 (A) | oli3347/oli3353 (A) |
|  |  |  | pMP3930 (B) | oli3352/oli3355 (B) |
|  |  |  | pCAMBIA1302^b^ (C) | oli3354/oli3351 (C) |
|  |  |  | PCR2: Fragments A to E from PCR1 | oli3347/oli3351 |
| pMP4028 | ALA1 genomic DNA | pENTR™/D-TOPO® | pMP3930 | oli3178/oli2096 |
| pMP4030 | ALA1 genomic DNA (no STOP codon) | pENTR™/D-TOPO® | pMP3930 | oli3178/oli1967B |

^a^Poulsen et al. 2008

^b^CAMBIA, Brisbane, Australia
