# Supplementary material for: A Putative Plant Aminophospholipid Flippase, the Arabidopsis P4 ATPase ALA1, Localizes to the Plasma Membrane following Association with a β-Subunit
Source: PLoS One. 2012 Apr 13;7(4):e33042. doi: 10.1371/journal.pone.0033042 (PMC3326016; doi:10.1371/journal.pone.0033042)
Supplement: Table S3 — Plasmids generated for expression of ALA1 and ALIS proteins in S. cerevisiae . ALA1 no TT: modified ALA1 gene in which a predicted transcription termination signal has been deleted; yeALA1: modified ALA1 gene in which a predicted transcription termination signal has been deleted and several codons codifying for arginine have been substituted to match the yeast preferred codon usage (see Materials and Methods and Figure S2). (DOC) [file pone.0033042.s007.doc]

| ALA1 cDNA cloning into yeast expression plasmids | | |
| --- | --- | --- |
| Plasmid name | Insert | Backbone (reference) |
| pMP2023 | ALA1 | pMP1965 (Poulsen et al. 2008) |
| pMP2024 | ALA1 + RGSH6::ALIS1 | pMP1965 (Poulsen et al. 2008) |
| pMP2026 | ALA1 + RGSH6::ALIS3 | pMP1965 (Poulsen et al. 2008) |
| pMP2028 | ALA1 + RGSH6::ALIS5 | pMP1965 (Poulsen et al. 2008) |
| pMP2434 | HA::ALA1 | pMP1965 (Poulsen et al. 2008) |
| pMP2435 | HA::ALA1 + RGSH6::ALIS1 | pMP1965 (Poulsen et al. 2008) |
| pMP2436 | HA::ALA1 + RGSH6::ALIS3 | pMP1965 (Poulsen et al. 2008) |
| pMP2437 | HA::ALA1 + RGSH6::ALIS5 | pMP1965 (Poulsen et al. 2008) |
| pMP3560 | (-3A)HA::ALA1 | pMP1965 (Poulsen et al. 2008) |
| pMP3561 | (-3A)HA::ALA1 + RGSH6::ALIS1 | pMP1965 (Poulsen et al. 2008) |
| pMP3562 | (-3A)HA::ALA1 + RGSH6::ALIS3 | pMP1965 (Poulsen et al. 2008) |
| pMP3563 | (-3A)HA::ALA1 + RGSH6::ALIS5 | pMP1965 (Poulsen et al. 2008) |
| pMP3635 | ALA1 no TT | pMP1965 (Poulsen et al. 2008) |
| pMP3636 | ALA1 no TT + RGSH6::ALIS1 | pMP1965 (Poulsen et al. 2008) |
| pMP3637 | ALA1 no TT + RGSH6::ALIS3 | pMP1965 (Poulsen et al. 2008) |
| pMP3638 | ALA1 no TT + RGSH6::ALIS5 | pMP1965 (Poulsen et al. 2008) |
| pMP3647 | HA::ALA1 no TT | pMP1965 (Poulsen et al. 2008) |
| pMP3648 | HA::ALA1 no TT + RGSH6::ALIS1 | pMP1965 (Poulsen et al. 2008) |
| pMP3649 | HA::ALA1 no TT + RGSH6::ALIS3 | pMP1965 (Poulsen et al. 2008) |
| pMP3650 | HA::ALA1 no TT + RGSH6::ALIS5 | pMP1965 (Poulsen et al. 2008) |
| pMP4070 | yeALA1 | pMP4062 (this study) |
| pMP4073 | ALA1 | pMP4062 (this study) |
| pMP4074 | ALA1 no TT | pMP4062 (this study) |
| pMP4079 | yeALA1 | pMP4075 (this study) |
| pMP2379 | RGSH6::ALIS1 | pRS426-Gal (Burgers et al. 1999) |
| pMP2381 | RGSH6::ALIS3 | pRS426-Gal (Burgers et al. 1999) |
| pMP2382 | RGSH6::ALIS5 | pRS426-Gal (Burgers et al. 1999) |
