# Supplementary material for: A Putative Plant Aminophospholipid Flippase, the Arabidopsis P4 ATPase ALA1, Localizes to the Plasma Membrane following Association with a β-Subunit
Source: PLoS One. 2012 Apr 13;7(4):e33042. doi: 10.1371/journal.pone.0033042 (PMC3326016; doi:10.1371/journal.pone.0033042)
Supplement: Table S4 — Plasmids for expression of fluorescently-tagged ALA1 in planta. (DOC) [file pone.0033042.s008.doc]

| Plasmid name | Insert | Backbone |
| --- | --- | --- |
| pMP3933 | ALA1 5’UTRs::ALA1 genomic DNA::GFP | pCAMBIA1302 (CAMBIA, Brisbane; Australia) |
| pMP4029 | GFP::ALA1 genomic DNA | pMPD43 (Curtis and Grossniklaus, 2003) |
| pMP4071 | ALA1 genomic DNA::GFP | pMPD83 (Curtis and Grossniklaus, 2003) |
